# Supplementary material for: Long-Term Calculation of Predicted Environmental Concentrations to Assess the Risk of Anticancer Drugs in Environmental Waters
Source: Molecules. 2022 May 17;27(10):3203. doi: 10.3390/molecules27103203 (PMC9147345; doi:10.3390/molecules27103203)
Supplement: Supplementary file 1 [file molecules-27-03203-s001.zip › molecules-1699046-supplementary.pdf]

Article

Long-term calculation of Predicted Environmental Concentrations to assess the risk of anticancer drugs in environmental waters

Pol Dominguez-García[1], Marta Gibert[1], Sílvia Lacorte[2] and Cristian Gómez-Canela[1,*]

[1] Department of Analytical and Applied Chemistry (Chromatography section), School of Engineering, Institut Químic de Sarrià-Universitat Ramon Llull, Via Agusta 390, 08017 Barcelona, Spain

[2] Institute for Environmental Assessment and Water Research (IDAEA-CSIC). Jordi Girona, 18. 08034 Barcelona, Spain

* Cristian Gómez-Canela, Institut Químic de Sarrià-Universitat Ramon Llull, Via Agusta 390, 08017 Barcelona, Spain. Telephone: +34-932672343. E-mail: cristian.gomez@iqs.url.edu

Table S1. Consumption data (t) of cytostatic families (ATC codes) from 2013 to 2017 period.

	**2013**	**2014**	**2015**	**2016**	**2017**
G03- Sex hormones	0.021	0.019	0.018	0.018	0.018
H02- Corticosteroids	0.24	0.25	0.29	0.33	0.35
L01- Antineoplastic agents	1.64	1.63	1.72	1.78	1.87
L02- Endocrine therapy	0.42	0.41	0.41	0.43	0.43
L03-- Immunostimulants	0.01	0.01	0.0004	0	0
L04- Immunosuppressants	2.57	2.77	2.88	2.99	3.1
TOTAL	4.89	5.1	5.33	5.55	5.76

Table S2. Levels of excretion and the percentage of removal for each cytostatic drug. F excretion is the contribution of both urine and feces excretion. Nd (no data avaiavle)

ATC code	Name	F excretion	Ref	Fwwtp removal	Ref
G03HA01	Cyproterone	0.33	[1]	0.15	[2]
H02AB07	Prednisone	0.5	nd	0.87	[2]
L01AA01	Cyclophosphamide	0.25	[3]	0.00	[4]
L01AA02	Chlorambucil	0.01	[5]	0.02	[2]
L01AA03	Melphalan	0.16	[3]	0.02	[2]
L01AA06	Ifosfamida	0.5	[4]	0.00	[4]
L01AA09	Bendamustine	0.05	[6]	0.16	[2]
L01AB01	Busulfan	0.02	[7]	0.02	[2]
L01AD02	Lomustine	0	[5]	0.04	[2]
L01AD04	Streptozocin	0.1	[7]	0.019	[2]
L01AD05	Fotemustine	0.5	nd	0.019	[2]
L01AX03	Temozolomide	0.38	[3]	0.0185	[2]
L01AX04	Dacarbazine	0.5	[4]	0.02	[2]
L01BA01	Methotrexate	0.9	[4]	0.95	[4]
L01BA03	Raltitrexed	0.5	[7]	0.0191	[2]
L01BA04	Pemetrexed	0.9	[3]	0.0185	[2]
L01BB02	Mercaptopurine	0.4	[7]	0.0186	[2]
L01BB03	Tioguanine	0.54	[7]	0.0185	[2]
L01BB04	Cladribine	0.44	[7]	0.0185	[2]
L01BB05	Fludarabine	0.6	[8]	0.0185	[2]
L01BB06	Clofarabine	0.6	[5]	0.0185	[2]
L01BB07	Nelarabine	0.066	[9]	0.0185	[2]
L01BC01	Cytarabine	0.1	[4]	0.50	[4]
L01BC02	Fluorouracil	0.39	[3]	0.90	[4]
L01BC03	Tegafur	0.05	[5]	0.02	[2]
L01BC05	Gemcitabine	0.1	[7]	0.40	[4]
L01BC06	Capecitabine	0.11	[8]	0.15	[4]
L01BC07	Azacitidine	0.85	[7]	0.02	[2]
L01BC53	Tegafur, combinations	0.5	nd	0.00	nd
L01CA01	Vinblastine	0.01	[7]	0.18	[2]
L01CA02	Vincristine	0.1	[7]	0.07	[2]
L01CA04	Vinorelbine	0.6	[3]	0.70	[2]
L01CA05	Vinflunine	0.33	[5]	0.87	[2]
L01CB01	Etoposide	0.93	[3]	0.0186	[2]
L01CD01	Paclitaxel	0.85	[3]	0.0938	[2]
L01CD02	Docetaxel	0.81	[3]	0.0448	[2]
L01CD04	Cabazitaxel	0.023	[5]	0.2257	[2]

L01CX01	Trabectedin	0.01	[5]	0.0212	[2]
L01DB01	Doxorubicin	0.5	[4]	0.0192	[2]
L01DB03	Epirubicin	0.1	[7]	0.0192	[2]
L01DB06	Idarubicin	0.07	[7]	0.0235	[2]
L01DB07	Mitoxantrone	0.23	[7]	0.026	[2]
L01DC01	Bleomycin	0.7	[7]	0.00	nd
L01DC03	Mitomycin	0.1	[4]	0.02	[2]
L01XA01	Cisplatin	0.6	[8]	0.02	[2]
L01XA02	Carboplatin	1	[4]	0.59	[10]
L01XA03	Oxaliplatin	0.5	[7]	0.00	nd
L01XC02	Rituximab	0.5	nd	0.00	nd
L01XC03	Trastuzumab	0.5	nd	0.00	nd
L01XC04	Alemtuzumab	0.5	nd	0.00	nd
L01XC06	Cetuximab	0.5	nd	0.00	nd
L01XC07	Bevacizumab	0.5	nd	0.00	nd
L01XC08	Panitumumab	0.5	nd	0.00	nd
L01XD03	Methyl aminolevulinate	0.5	nd	0.02	[2]
L01XD04	Aminolevulinic acid	0.5	nd	0.02	[2]
L01XE01	Imatinib	0.25	[4]	0.06	[2]
L01XE02	Gefitinib	0.05	[4]	0.19	[2]
L01XE03	Erlotinib	0.02	[4]	0.04	[2]
L01XE04	Sunitinib	0.77	[3]	0.04	[2]
L01XE05	Sorafenib	0.51	[5]	0.85	[2]
L01XE06	Dasatinib	0.19	[7]	0.02	[2]
L01XE07	Lapatinib	0.67	[5]	0.85	[2]
L01XE08	Nilotinib	0.69	[7]	0.78	[2]
L01XE09	Temsirolimus	0.05	[7]	0.00	nd
L01XE10	Everolimus	0.85	[3]	0.00	nd
L01XE11	Pazopanib	0.82	[3]	0.08	[2]
L01XX05	Hydroxycarbamide	0.5	[4]	0.02	[2]
L01XX08	Pentostatin	0.9	[5]	0.02	[2]
L01XX09	Miltefosine	0.5	nd	0.12	[2]
L01XX11	Estramustine	0.05	[4]	0.88	[2]
L01XX14	Tretinoin	0.63	[7]	0.93	[2]
L01XX17	Topotecan	0.66	[5]	0.02	[2]
L01XX19	Irinotecan	0.5	[4]	0.03	[2]
L01XX23	Mitotane	0.6	[4]	0.92	[2]
L01XX25	Bexarotene	0.01	[7]	0.94	[2]
L01XX27	Arsenic trioxide	0.15	[5]	0.02	[2]
L01XX32	Bortezomib	0.5	nd	0.02	[2]
L01XX33	Celecoxib	0.01	[5]	0.12	[2]
L01XX35	Anagrelide	0.01	[5]	0.02	[2]
L02AA01	Dietilestilbestrol	0.5	nd	0.80	[2]

L02AA04	Fosfestrol	0.5	nd	0.67	[2]
L02AB01	Megestrol	0.78	[7]	0.96	[11]
L02AB02	Medroxyprogesterone	0.5	nd	0.13	[2]
L02AE01	Buserelin	0.2	[7]	0.02	[2]
L02AE02	Leuprorelin	0.05	[7]	0.02	[2]
L02AE03	Goserelin	0.9	[1]	0.00	nd
L02AE04	Triptorelin	0.42	[5]	0.00	nd
L02BA01	Tamoxifen	0.5	[3]	0.93	[2]
L02BA02	Toremifene	0.5	nd	0.94	[2]
L02BA03	Fulvestrant	0.9	[3]	0.94	[2]
L02BB01	Flutamide	0.1	[4]	0.10	[2]
L02BB03	Bicalutamide	0.55	[4]	0.03	[2]
L02BG03	Anastrozole	0.1	[12]	0.03	[2]
L02BG04	Letrozole	0.06	[12]	0.03	[2]
L02BG06	Exemestane	0.01	[12]	0.90	[4]
L02BX03	Abiraterone	0.93	[3]	0.87	[2]
L03AA02	Filgrastim	0.5	nd	0.00	nd
L03AA10	Lenograstim	0.01	[5]	0.00	nd
L03AA13	Pegfilgrastim	0.5	nd	0.00	nd
L03AB03	Interferon gamma	0.5	nd	0.00	nd
L03AB04	Interferon alfa-2a	0.5	nd	0.00	nd
L03AB05	Interferon alfa-2b	0.5	nd	0.00	nd
L03AB07	Interferon beta-1a	0.5	nd	0.00	nd
L03AB08	Interferon beta-1b	0.5	nd	0.00	nd
L03AB10	Peginterferon alfa-2b	0.5	nd	0.00	nd
L03AB11	Peginterferon alfa-2a	0.5	nd	0.00	nd
L03AC01	Aldesleukin	0.5	nd	0.00	nd
L03AX03	BCG vaccine	0.5	nd	0.00	nd
L03AX13	Glatiramer, acetate	0.5	nd	0.00	nd
L03AX16	Plerixafor	0.7	[5]	0.02	[2]
L04AA04	Antithymocyte immunoglobulin (rabbit)	0.5	nd	0.00	nd
L04AA06	Mycophenolic acid	0.63	[9]	0.41	[2]
L04AA10	Sirolimus	0.02	[9]	0.71	[2]
L04AA13	Leflunomide	0.91	[3]	0.03	[2]
L04AA18	Everolimus	0.85	[3]	0.58	[2]
L04AA23	Natalizumab	0.5	nd	0.00	nd
L04AA24	Abatacept	0.5	nd	0.00	nd
L04AA25	Eculizumab	0.5	nd	0.00	nd
L04AA27	Fingolimod	0.025	[5]	0.80	[2]
L04AB01	Etanercept	0.5	nd	0.00	nd
L04AB02	Infliximab	0.5	nd	0.00	nd
L04AB04	Adalimumab	0.5	nd	0.00	nd
L04AB05	Certolizumab pegol	0.5	nd	0.00	nd

L04AB06	Golimumab	0.5	nd	0.00	nd
L04AC03	Anakinra	0.5	nd	0.00	nd
L04AC05	Ustekinumab	0.5	nd	0.00	nd
L04AC07	Tocilizumab	0.5	nd	0.00	nd
L04AC08	Canakinumab	0.5	nd	0.00	nd
L04AD01	Ciclosporin	0.01	[3]	0.00	nd
L04AD02	Tacrolimus	0.01	[9]	0.60	[2]
L04AX01	Azathioprine	0.02	[12]	0.02	[2]
L04AX04	Lenalidomide	0.94	[3]	0.02	[2]

Table S3. Consumption data, PEC_{wwtp} and PEC_{river} from all 132 cytostatic compounds from 2013 to 2017 period. Nq (non-quantificable)

		2013			2014			2015			2016			2017		
ATC code	Name	Consume total-g/day	PEC WWTP eff ng/L	PEC river ng/L	Consume total-g/day	PEC WWTP eff ng/L	PEC river ng/L	Consume total-g/day	PEC WWTP eff ng/L	PEC river ng/L	Consume total-g/day	PEC WWTP eff ng/L	PEC river ng/L	Consume total-g/day	PEC WWTP eff ng/L	PEC river ng/L
G03HA01	Cyproterone	56	16	0.61	53	15	0.57	50	14	0.55	50	14	0.55	49	14	0.54
H02AB07	Prednisone	660	43	1.7	690	45	1.8	780	51	2	900	59	2.3	960	63	2.4
L01AA01	Cyclophosphamide	12	3	0.11	12	3.1	0.12	11	2.8	0.11	13	3.4	0.13	22	5.5	0.21
L01AA02	Chlorambucil	0.47	0.0046	0.00018	0.23	0.0023	0.000088	0.00055	0.000054	0.0000021	nq	nq	nq	nq	nq	nq
L01AA03	Melphalan	0.28	0.045	0.0017	0.14	0.022	0.00086	0.00027	0.000043	0.0000017	nq	nq	nq	nq	nq	nq
L01AA06	Ifosfamida	19	9.4	0.36	20	10	0.39	20	10	0.4	18	9.1	0.35	17	8.8	0.34
L01AA09	Bendamustine	0.27	0.011	0.00044	0.37	0.016	0.0006	0.49	0.021	0.00081	0.52	0.022	0.00085	0.44	0.019	0.00072
L01AB01	Busulfan	0.018	0.00036	0.000014	0.011	0.00022	0.0000085	0.0022	0.000043	0.0000017	nq	nq	nq	nq	nq	nq
L01AD02	Lomustine	nq	nq	nq	nq	nq	nq	nq	nq	nq	nq	nq	nq	nq	nq	nq
L01AD04	Streptozocin	1	0.1	0.0039	1.5	0.14	0.0055	nq	nq	nq	nq	nq	nq	nq	nq	nq

L01AD05	Fotemustine	0.038	0.019	0.00073	0.047	0.023	0.0009	0.19	0.096	0.0037	0.22	0.11	0.0041	0.24	0.12	0.0047
L01AX03	Temozolomide	9.9	3.7	0.14	9.3	3.5	0.13	9.5	3.6	0.14	11	4.1	0.16	12	4.4	0.17
L01AX04	Dacarbazine	0.13	0.065	0.0025	0.06	0.03	0.0012	0.041	0.02	0.00079	0.0027	0.0014	0.000052	0.012	0.0061	0.00024
L01BA01	Methotrexate	28	1.3	0.048	29	1.3	0.051	31	1.4	0.054	1.7	0.079	0.003	2.3	0.1	0.004
L01BA03	Raltitrexed	0.0038	0.0019	0.000072	nq	nq	nq	0.0057	0.0028	0.00011	0.0061	0.003	0.00012	0.0058	0.0029	0.00011
L01BA04	Pemetrexed	10	9.3	0.36	12	10	0.4	13	12	0.46	13	12	0.45	12	11	0.41
L01BB02	Mercaptopurine	18	7	0.27	11	4.2	0.16	0.027	0.011	0.00042	14	5.7	0.22	26	10	0.4
L01BB03	Tioguanine	0.047	0.025	0.00096	0.055	0.029	0.0011	nq	nq	nq	nq	nq	nq	nq	nq	nq
L01BB04	Cladribine	nq	nq	nq	0.01	0.0044	0.00017	0.0036	0.0016	0.00006	0.0076	0.0033	0.00013	0.0039	0.0017	0.000065
L01BB05	Fludarabine	0.19	0.11	0.0043	0.17	0.1	0.0038	0.17	0.1	0.004	0.17	0.1	0.004	0.13	0.079	0.0031
L01BB06	Clofarabine	0.00055	0.00033	0.000013	0.0019	0.0011	0.000043	0.0011	0.00065	0.000025	nq	nq	nq	nq	nq	nq
L01BB07	Nelarabine	2.8	0.18	0.007	3.7	0.24	0.0093	nq	nq	nq	0.014	0.0009	0.000035	nq	nq	nq
L01BC01	Cytarabine	0.0038	0.00019	0.0000075	nq	nq	nq	4.2	0.21	0.0082	4.2	0.21	0.0082	10	0.52	0.02
L01BC02	Fluorouracil	2.3	0.09	0.0035	2.5	0.097	0.0037	1.3	0.052	0.002	1.4	0.055	0.0021	0.36	0.014	0.00054
L01BC03	Tegafur	41	2	0.079	30	1.5	0.058	27	1.3	0.052	18	0.87	0.033	14	0.69	0.027
L01BC05	Gemcitabine	87	5.2	0.2	92	5.6	0.22	92	5.6	0.22	92	5.6	0.21	94	5.7	0.22
L01BC06	Capecitabine	1800	170	6.4	1600	150	5.9	1800	170	6.4	1800	170	6.5	1900	180	6.9
L01BC07	Azacitidine	0.9	0.76	0.029	1.1	0.95	0.037	4.8	4	0.16	5.3	4.5	0.17	6.2	5.2	0.2
L01BC53	Tegafur, combinations	nq	nq	nq	nq	nq	nq	nq	nq	nq	nq	nq	nq	nq	nq	nq
L01CA01	Vinblastine	0.000082	0.00000068	2.6E-08	nq	nq	nq	0.00082	0.0000068	0.00000026	0.000082	0.00000068	2.6E-08	0.0011	0.00009	3.5E-08
L01CA02	Vincristine	0.000066	0.0000062	0.00000024	0.00028	0.000027	0.000001	0.000071	0.0000067	0.00000026	0.000055	0.0000052	0.0000002	0.0071	0.00067	0.000026

L01CA 04	Vinorelbine	1.5	0.27	0.01	13	2.4	0.092	1.4	0.25	0.0097	1.3	0.23	0.0088	1.4	0.26	0.0099
L01CA 05	Vinflunine	nq	nq	nq	nq	nq	nq	nq	nq	nq	nq	nq	nq	nq	nq	nq
L01CB 01	Etoposide	0.58	0.53	0.02	0.44	0.41	0.016	0.5	0.46	0.018	4.2	3.9	0.15	4.4	4.1	0.16
L01CD 01	Paclitaxel	13	10	0.4	16	13	0.49	19	15	0.57	21	17	0.64	25	19	0.75
L01CD 02	Docetaxel	2.8	2.2	0.085	1.5	1.2	0.046	3.3	2.6	0.099	2.8	2.2	0.084	2.3	1.8	0.069
L01CD 04	Cabazitaxel	0.024	0.00043	0.00001 6	0.04 8	0.000 87	0.00003 3	0.064	0.0011	0.00004 4	0.065	0.0012	0.00004 5	0.08 1	0.0015	0.00005 6
L01CX 01	Trabectedin	0.001 3	0.00001 3	0.00000 05	0.00 12	0.000 011	0.00000 044	0.001 4	0.0000 14	0.00000 054	0.001 7	0.00001 7	0.00000 066	0.00 12	0.0000 12	0.00000 047
L01DB 01	Doxorubicin	0.24	0.12	0.0046	0.26	0.13	0.0049	0.46	0.23	0.0087	0.49	0.24	0.0093	0.5	0.25	0.0096
L01DB 03	Epirubicin	0.062	0.0061	0.00023	0.03 7	0.003 7	0.00014	0.015	0.0015	0.00005 8	0.019	0.0019	0.00007 2	0.00 16	0.0001 6	0.00000 63
L01DB 06	Idarubicin	0.001 7	0.00011	0.00000 44	nq	nq	nq	nq	nq	nq	nq	0.069	0.0027	0.00 018	0.0000 12	0.00000 047
L01DB 07	Mitoxantrone	0.000 27	0.00006 2	0.00000 24	nq	nq	nq	nq	nq	nq	nq	nq	nq	nq	nq	nq
L01DC 01	Bleomycin	0.001 1	0.00075	0.00002 9	nq	nq	nq	nq	nq	nq	0.076	0.054	0.0021	0.07 2	0.051	0.002
L01DC 03	Mitomycin	0.058	0.0057	0.00022	0.06 3	0.006 3	0.00024	0.074	0.0073	0.00028	0.041	0.0041	0.00016	0.01 3	0.0013	0.00005
L01XA 01	Cisplatin	0.92	0.54	0.021	0.83	0.49	0.019	4.3	2.6	0.099	4.4	2.6	0.1	4.4	2.6	0.1
L01XA 02	Carboplatin	0.19	0.079	0.003	0.15	0.062	0.0024	0.1	0.043	0.0017	0.1	0.041	0.0016	0.07 1	0.03	0.0011
L01XA 03	Oxaliplatin	3.2	1.6	0.063	2.9	1.4	0.056	9.5	4.8	0.18	10	5.3	0.2	11	5.7	0.22
L01XC 02	Rituximab	18	9	0.35	19	9.5	0.37	20	9.9	0.38	24	12	0.46	30	15	0.58
L01XC 03	Trastuzumab	15	7.7	0.3	16	8	0.31	19	9.4	0.36	23	12	0.45	24	12	0.47
L01XC 04	Alemtuzumab	nq	nq	nq	nq	nq	nq	nq	nq	nq	nq	nq	nq	nq	nq	nq
L01XC 06	Cetuximab	13	6.7	0.26	1.3	0.64	0.025	12	5.8	0.22	13	6.7	0.26	13	6.7	0.26
L01XC 07	Bevacizumab	8.8	4.4	0.17	8.5	4.3	0.17	10	5.1	0.2	12	5.9	0.23	14	6.8	0.26

L01XC08	Panitumumab	1.9	0.97	0.037	2.2	1.1	0.043	2.3	1.2	0.045	3.2	1.6	0.063	2.7	1.4	0.053
L01XD03	Methyl aminolevulinate	0.76	0.38	0.015	0.79	0.39	0.015	1.1	0.56	0.022	1.6	0.81	0.031	2.1	1	0.04
L01XD04	Aminolevulinic acid	0.00085	0.00042	0.000016	0.009	0.0044	0.00017	0.059	0.029	0.0011	0.16	0.079	0.0031	0.34	0.17	0.0065
L01XE01	Imatinib	270	65	2.5	280	67	2.6	270	64	2.5	280	67	2.6	300	71	2.7
L01XE02	Gefitinib	20	0.8	0.031	17	0.68	0.026	17	0.68	0.026	17	0.68	0.026	15	0.59	0.023
L01XE03	Erlotinib	18	0.35	0.014	18	0.35	0.014	15	0.3	0.011	13	0.26	0.0099	11	0.21	0.0082
L01XE04	Sunitinib	3.4	2.5	0.097	3.3	2.5	0.096	3.5	2.6	0.1	3.5	2.6	0.1	3.7	2.7	0.11
L01XE05	Sorafenib	64	4.9	0.19	65	5	0.19	66	5.1	0.2	82	6.3	0.24	80	6.1	0.24
L01XE06	Dasatinib	9.6	1.8	0.07	10	1.9	0.075	18	3.4	0.13	15	2.8	0.11	15	2.8	0.11
L01XE07	Lapatinib	66	6.7	0.26	63	6.4	0.25	49	5	0.19	34	3.4	0.13	32	3.2	0.12
L01XE08	Nilotinib	77	12	0.45	82	13	0.48	81	12	0.48	93	14	0.55	93	14	0.55
L01XE09	Temsirolimus	0.06	0.003	0.00012	0.046	0.0023	0.000089	0.041	0.0021	0.00008	0.037	0.0018	0.000071	0.024	0.0012	0.000047
L01XE10	Everolimus	0.09	0.077	0.003	0.4	0.34	0.013	0.61	0.53	0.02	0.81	0.69	0.027	0.92	0.79	0.03
L01XE11	Pazopanib	24	18	0.69	39	30	1.1	37	28	1.1	41	31	1.2	41	31	1.2
L01XX05	Hydroxycarbamide	1800	890	34	1900	940	36	2000	990	38	2100	1100	41	2200	1100	42
L01XX08	Pentostatin	0.0012	0.0011	0.000041	0.0014	0.0012	0.000048	0.00099	0.00088	0.000034	0.00044	0.00039	0.000015	0.0013	0.0011	0.000043
L01XX09	Miltefosine	nq	nq	nq	nq	nq	nq	nq	nq	nq	nq	nq	nq	nq	nq	nq
L01XX11	Estramustine	15	0.091	0.0035	12	0.07	0.0027	5.6	0.033	0.0013	4.1	0.024	0.00093	1.5	0.0091	0.00035
L01XX14	Tretinoin	0.68	0.03	0.0012	0.65	0.029	0.0011	0.79	0.035	0.0013	0.82	0.036	0.0014	0.92	0.04	0.0016
L01XX17	Topotecan	0.024	0.016	0.0006	0.021	0.014	0.00054	0.019	0.013	0.00049	0.017	0.011	0.00042	0.017	0.011	0.00043
L01XX19	Irinotecan	11	5.3	0.21	8.7	4.3	0.16	13	6.6	0.25	13	6.2	0.24	13	6.4	0.25

L01XX																
23	Mitotane	30	1.5	0.059	40	2.1	0.08	40	2.1	0.079	41	2.1	0.08	35	1.8	0.07
L01XX						0.004										
25	Bexarotene	6.1	0.0037	0.00014	6.9	2	0.00016	8.1	0.0049	0.00019	8.7	0.0053	0.0002	6.9	0.0041	0.00016
L01XX		0.003		0.00001	0.00	0.001	0.00004	0.005	0.0007	0.00002			0.00006	0.01		
27	Arsenic trioxide	3	0.00048	9	81	2	7	1	6	9	0.011	0.0016	3	2	0.0018	0.00007
L01XX					0.06									0.08		
32	Bortezomib	0.061	0.03	0.0012	6	0.033	0.0013	0.08	0.04	0.0015	0.091	0.045	0.0017	8	0.043	0.0017
L01XX																
33	Celecoxib	nq	nq	nq	nq	nq	nq	nq	nq	nq	nq	nq	nq	nq	nq	nq
L01XX						0.004										
35	Anagrelide	0.46	0.0046	0.00018	0.49	9	0.00019	0.48	0.0047	0.00018	0.47	0.0047	0.00018	0.48	0.0048	0.00018
L02AA		0.002		0.00000	0.00	0.000	0.00000									
01	Dietilestilbestrol	1	0.00021	81	18	18	7	nq	nq	nq	nq	nq	nq	nq	nq	nq
L02AA																
04	Fosfestrol	nq	nq	nq	nq	nq	nq	nq	nq	nq	nq	nq	nq	nq	nq	nq
L02AB																
01	Megestrol	450	14	0.54	400	12	0.48	390	12	0.48	470	15	0.57	450	14	0.54
L02AB																
02	Medroxyprogesterone	9.7	4.2	0.16	9.8	4.3	0.17	11	4.7	0.18	12	5.3	0.2	10	4.5	0.17
L02AE					0.01	0.002	0.00009			0.00007			0.00005	0.00		0.00004
01	Buserelin	0.015	0.0031	0.00012	3	5	7	0.01	0.002	7	0.007	0.0014	4	59	0.0012	5
L02AE																
02	Leuprorelin	1.4	0.069	0.0027	1.3	0.065	0.0025	1.3	0.063	0.0024	1.6	0.081	0.0031	1.7	0.083	0.0032
L02AE					0.09									0.09		
03	Goserelin	0.12	0.11	0.0041	6	0.088	0.0034	0.091	0.083	0.0032	0.091	0.083	0.0032	7	0.088	0.0034
L02AE																
04	Triptorelin	0.7	0.3	0.012	0.72	0.31	0.012	0.76	0.32	0.012	0.89	0.38	0.015	0.91	0.39	0.015
L02BA																
01	Tamoxifen	140	5	0.19	150	5.2	0.2	150	5.2	0.2	140	5	0.19	140	5	0.19
L02BA																
02	Toremifene	nq	nq	nq	nq	nq	nq	nq	nq	nq	nq	nq	nq	nq	nq	nq
L02BA																
03	Fulvestrant	0.85	0.046	0.0018	0.93	0.05	0.0019	1.1	0.062	0.0024	5.1	0.28	0.011	6.7	0.36	0.014
L02BB																
01	Flutamide	120	11	0.42	99	9	0.35	78	7.1	0.27	67	6.1	0.24	47	4.3	0.16
L02BB																
03	Bicalutamide	240	130	5.1	220	120	4.6	200	110	4.2	180	100	3.8	170	90	3.5
L02BG																
03	Anastrozole	2.5	0.24	0.0094	2.1	0.21	0.0081	1.9	0.19	0.0072	1.8	0.18	0.0068	1.8	0.17	0.0067
L02BG																
04	Letrozole	20	1.2	0.047	22	1.3	0.051	24	1.4	0.055	27	1.6	0.061	30	1.7	0.067

L02BG06	Exemestane	51	0.051	0.002	51	0.051	0.002	50	0.05	0.0019	48	0.049	0.0019	48	0.049	0.0019
L02BX03	Abiraterone	110	13	0.51	180	22	0.85	230	28	1.1	230	28	1.1	270	33	1.3
L03AA02	Filgrastim	2.1	1.1	0.042	0.86	0.44	0.017	nq	nq	nq	nq	nq	nq	nq	nq	nq
L03AA10	Lenograstim	2.1	0.022	0.00084	0.86	0.0087	0.00034	nq	nq	nq	nq	nq	nq	nq	nq	nq
L03AA13	Pegfilgrastim	0.046	0.023	0.0009	0.033	0.017	0.00064	nq	nq	nq	nq	nq	nq	nq	nq	nq
L03AB03	Interferon gamma	0.00027	0.00014	0.0000052	0.00017	0.000086	0.0000033	0.00014	0.000069	0.0000027	nq	nq	nq	nq	nq	nq
L03AB04	Interferon alfa-2a	0.000018	0.0000091	0.00000035	0.00003	0.000015	0.00000058	0.00003	0.000015	0.00000058	nq	nq	nq	nq	nq	nq
L03AB05	Interferon alfa-2b	0.000013	0.0000065	0.00000025	0.00008	0.00004	0.0000016	0.000082	0.000041	0.0000016	nq	nq	nq	nq	nq	nq
L03AB07	Interferon beta-1a	0.017	0.0083	0.00032	0.016	0.0081	0.00031	nq	nq	nq	nq	nq	nq	nq	nq	nq
L03AB08	Interferon beta-1b	nq	nq	nq	0.098	0.05	0.0019	nq	nq	nq	nq	nq	nq	nq	nq	nq
L03AB10	Peginterferon alfa-2b	0.0033	0.0017	0.000065	0.0026	0.0013	0.00005	nq	nq	nq	nq	nq	nq	nq	nq	nq
L03AB11	Peginterferon alfa-2a	0.017	0.0084	0.00032	0.015	0.0077	0.0003	nq	nq	nq	nq	nq	nq	nq	nq	nq
L03AC01	Aldesleukin	0.00034	0.00017	0.0000067	nq	nq	nq	nq	nq	nq	nq	nq	nq	nq	nq	nq
L03AX03	BCG vaccine	2.4	1.2	0.046	2.3	1.2	0.045	1.2	0.6	0.023	nq	nq	nq	nq	nq	nq
L03AX13	Glatiramer, acetate	11	5.6	0.21	12	6	0.23	nq	nq	nq	nq	nq	nq	nq	nq	nq
L03AX16	Plerixafor	0.0025	0.0017	0.000066	0.0033	0.0023	0.000088	nq	nq	nq	nq	nq	nq	nq	nq	nq
L04AA04	Antithymocyte immuno-globulin (rabbit)	0.00033	0.00017	0.0000064	0.0003	0.00015	0.0000059	nq	nq	nq	nq	nq	nq	nq	nq	nq
L04AA06	Mycophenolic acid	5900	2200	86	6500	2400	93	6800	2500	98	7000	2600	100	7300	2700	110
L04AA10	Sirolimus	0.95	0.0062	0.00024	1.1	0.007	0.00027	1.1	0.0072	0.00028	1.1	0.0074	0.00029	1.2	0.0077	0.0003
L04AA13	Leflunomide	47	42	1.6	50	45	1.7	52	46	1.8	55	49	1.9	57	51	2
L04AA18	Everolimus	1.1	0.41	0.016	1.3	0.46	0.018	1.4	0.49	0.019	1.5	0.55	0.021	1.6	0.59	0.023

L04AA23	Natalizumab	3.7	1.8	0.071	3.6	1.8	0.069	nq	nq	nq	nq	nq	nq	nq	nq	nq
L04AA24	Abatacept	7.1	3.6	0.14	4.5	2.3	0.088	nq	nq	nq	nq	nq	nq	nq	nq	nq
L04AA25	Eculizumab	0.84	0.43	0.016	1.3	0.66	0.025	1.6	0.79	0.031	1.9	0.95	0.037	2.5	1.3	0.049
L04AA27	Fingolimod	0.087	0.00043	0.000017	0.16	0.00078	0.00003	nq	nq	nq	nq	nq	nq	nq	nq	nq
L04AB01	Etanercept	16	7.9	0.31	16	8	0.31	nq	nq	nq	nq	nq	nq	nq	nq	nq
L04AB02	Infliximab	13	6.5	0.25	14	6.8	0.26	nq	nq	nq	nq	nq	nq	nq	nq	nq
L04AB04	Adalimumab	8.6	4.3	0.17	9.2	4.6	0.18	nq	nq	nq	nq	nq	nq	nq	nq	nq
L04AB05	Certolizumab pegol	2.6	1.3	0.052	3.7	1.8	0.071	nq	nq	nq	nq	nq	nq	nq	nq	nq
L04AB06	Golimumab	0.46	0.23	0.0089	0.72	0.36	0.014	nq	nq	nq	nq	nq	nq	nq	nq	nq
L04AC03	Anakinra	4.1	2.1	0.08	6.1	3.1	0.12	nq	nq	nq	nq	nq	nq	nq	nq	nq
L04AC05	Ustekinumab	0.3	0.15	0.0058	0.4	0.2	0.0079	nq	nq	nq	nq	nq	nq	nq	nq	nq
L04AC07	Tocilizumab	6.3	3.2	0.12	7.3	3.7	0.14	nq	nq	nq	nq	nq	nq	nq	nq	nq
L04AC08	Canakinumab	0.0066	0.0033	0.00013	0.009	0.0046	0.00018	nq	nq	nq	nq	nq	nq	nq	nq	nq
L04AD01	Ciclosporin	300	3	0.11	290	2.9	0.11	280	2.8	0.11	270	2.7	0.11	250	2.5	0.095
L04AD02	Tacrolimus	20	0.082	0.0032	23	0.092	0.0035	25	0.1	0.0039	32	0.13	0.005	36	0.14	0.0055
L04AX01	Azathioprine	670	13	0.51	710	14	0.54	750	15	0.58	790	16	0.61	810	16	0.62
L04AX04	Lenalidomide	1.7	1.5	0.059	1.9	1.8	0.069	2	1.9	0.072	2.8	2.6	0.1	3.7	3.5	0.13
